# 

**Published:** 1888-08-04

**Authors:** 


**Nurse-training Schools and Registration** - - 285

**Metropolitan Hospital Sunday Fund, 1888** - - 286

**The Doctor's Pulpit.**—Human Barometers - - - 287

**Within the Wards.**—Dangerous Abuse of a New Drug—Two Bullets in a Patient's Brain, etc. - - - - - - 288

**Malingering** - - - - - - - 289

**Round About the Asylums**—XI. - - - - 290

**Hints for the Sick-room.**—By M. M. BASIL, M.A, M.B. 291

**Notes and News** - - - - - - 292

**Everybody's Page** - - - - - - 294

**Army Recollections.** - - - - - - 296

**Annotations.**—Paupers for Farmers.—Cottage Industries in Ireland.—The Progress of Self-help among Workmen - - 295

**An Ishmaelite.** By LESLIE KEITH, Author of "The Chilcotes," "East and West," etc. - - - - - - 297

**Difficult Cases** - - - - - - - 298

**New Remedies and Appliances** - - - - 299

**Lonely** - - - - - - - - 299

**The Editor's Letter-box** - - - - - 299

**The Dispenser's Column** - - - - - 299

**Amusements with Profit for all Ages.**—For Men and Women.—Children's Corner.—Puzzles, etc. - - - 300

**EXTRA SUPPLEMENT.—THE NURSING MIRROR.**

En Passant.—A Lady Dentist.—St. John's Sisterhood.—Poultices.—The Question of Uniform.—The Manchester Infirmary - lxix
Original Articles.—The Progress of Nursing in New York - - lxx
How to Spend a Half-holiday - - - - - - lxxi
Everybody's Opinion.—The Life of a Private Nurse.—The Wearing of a Uniform.—A Mistaken Vocation - - - lxxii
Appointments - - - - - - - - lxxii
Vacancies - - - - - - - - lxxii

